# Technical Approach to Laparoscopic Examination of the Small Bowel in Gallstone Ileus

**DOI:** 10.1155/2020/8852804

**Published:** 2020-10-10

**Authors:** Sarah Assali, John Mourany, Brendan Jones, Lauren Dudas, Nova Szoka

**Affiliations:** ^1^Allegheny Health Network, USA; ^2^West Virginia University, USA

## Abstract

**Background:**

Gallstone ileus is an infrequent cause of small bowel obstructions (SBO), accounting for only 0.1-5% of SBOs and 25% of nonstrangulating causes of SBO in the elderly population. There is scant literature available regarding the use of laparoscopy to treat gallstone ileus. Currently, much of the literature available is limited to case reports only.

**Methods:**

A complete laparoscopic approach was utilized to manage a 65-year-old woman with morbid obesity who presented with gallstone ileus. With regard to our technical approach, we describe the technical approach that facilitates safe laparoscopic examination of the entire small bowel and can be applied to other acute care surgery cases involving small bowel pathology.

**Results:**

The patient's postoperative course was complicated by new-onset atrial fibrillation which was treated medically with good response. She was safely discharged on postoperative day 2.

**Conclusion:**

Laparoscopy is a feasible option for the management of gallstone ileus and can lead to decreased morbidity compared to laparotomy. The technique described allows for laparoscopic examination of the entire small bowel.

## 1. Introduction

Gallstone ileus is a condition that results when a biliary calculus gains entry to the intestinal lumen through a biliary-enteric fistula and causes mechanical bowel obstruction [1, 2]. Most commonly a cholecystoduodenal fistula, the fistula forms as a result of longstanding inflammation from cholelithiasis. As the gallstone travels through the small bowel, obstruction most commonly occurs in the terminal ileum but can be seen at other locations in the gastrointestinal tract [3]. While 25% to 72% of patients with gallstone ileus have a history of cholelithiasis, only 0.3% to 1.5% of patients with cholelithiasis will develop a gallstone ileus [1, 4]. Historically, gallstone ileus was thought to account for 1% to 5% of all small bowel obstructions and 25% of nonstrangulating small bowel obstructions in the elderly population [1, 5]. More recently, a review of the Nationwide Inpatient Sample database showed that gallstone ileus made up 0.095% of all bowel obstructions and tends to occur with higher prevalence in the elderly, female population [1].

Initial management is directed at fluid resuscitation and resolving electrolyte imbalances followed by surgery [6]. Classically, surgery has involved exploratory laparotomy with stone extraction via enterolithotomy [6]. The enterotomy is created in a longitudinal fashion and closed in a transverse fashion to prevent stenosis [7]. More recently, laparoscopy has been shown effective in the treatment of this disease [1, 7]. Regardless of operative approach, the remainder of the bowel should be examined to determine if other gallstones are present. Closure of the cholecystoduodenal fistula is not necessary at the initial operation; however, due to the low prevalence of the disease optimal management of the fistula, it remains uncertain [2, 3, 6].

Recent studies have shown that enterotomy and stone extraction alone are safe and that enterotomy with fistula closure or bowel resection was associated with higher mortality rates and greater length of stay [1, 4]. Despite operation, mortality still ranges from 15% to 18% and the process recurs in up to 5% to 17% of patients [3, 4, 6].

The purpose of this manuscript is to present the laparoscopic management of gallstone ileus, including a brief discussion of the operative technique for laparoscopic examination of the entire length of small bowel in order to demonstrate that this approach can be a safe alternative to open surgery and may to reduce morbidity, mortality, and hospital length of stay for this disease.

## 2. Methods

Written consent was obtained from the patient to use this case for educational purposes. The West Virginia Institutional Review Board approved this study (Protocol no. 2004964936). In this study, we portray the successful use of laparoscopy in the management of gallstone ileus in the case of a bariatric patient with multiple comorbidities. The patient was a 65-year-old woman with a past medical history of morbid obesity (BMI 38), obstructive sleep apnea with 2-liter nocturnal oxygen requirement, hypertension, GERD, active tobacco use, and urinary incontinence. Her past surgical history included placement of a cholecystostomy tube six years prior, with subsequent removal. The patient was an active tobacco user, smoking 1 pack per day, but did not use alcohol or recreational drugs. She had no known drug allergies and was on a single blood pressure medication, a proton pump inhibitor for reflux disease, and Ditropan for urinary incontinence.

**Figure 1:**
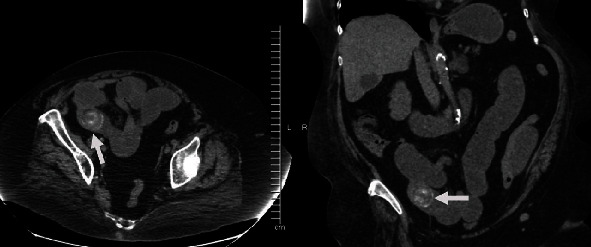
CT scan demonstrating large gallstone (white arrow) causing a small bowel obstruction.

The patient was transferred to our facility from an outside hospital with a 3-day history of abdominal pain, nausea, vomiting, and obstipation. She was found to have a leukocytosis of 20 k/mcL and CT scan findings consistent with pneumobilia, a small bowel obstruction, and an ectopic gallstone (Figure 1). She was consented for surgery and taken urgently for operative exploration. The operation was completed in an entirely laparoscopic fashion.

**Figure 2:**
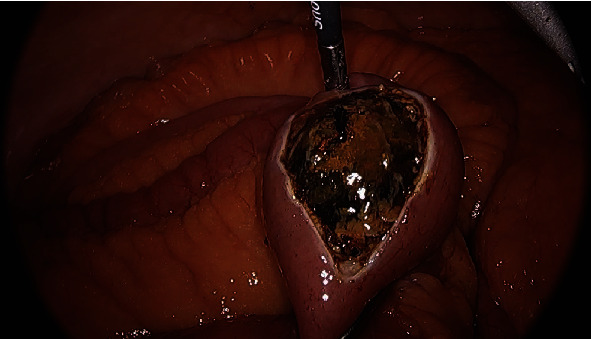
Enterotomy with large gallstone exposed.

**Figure 3:**
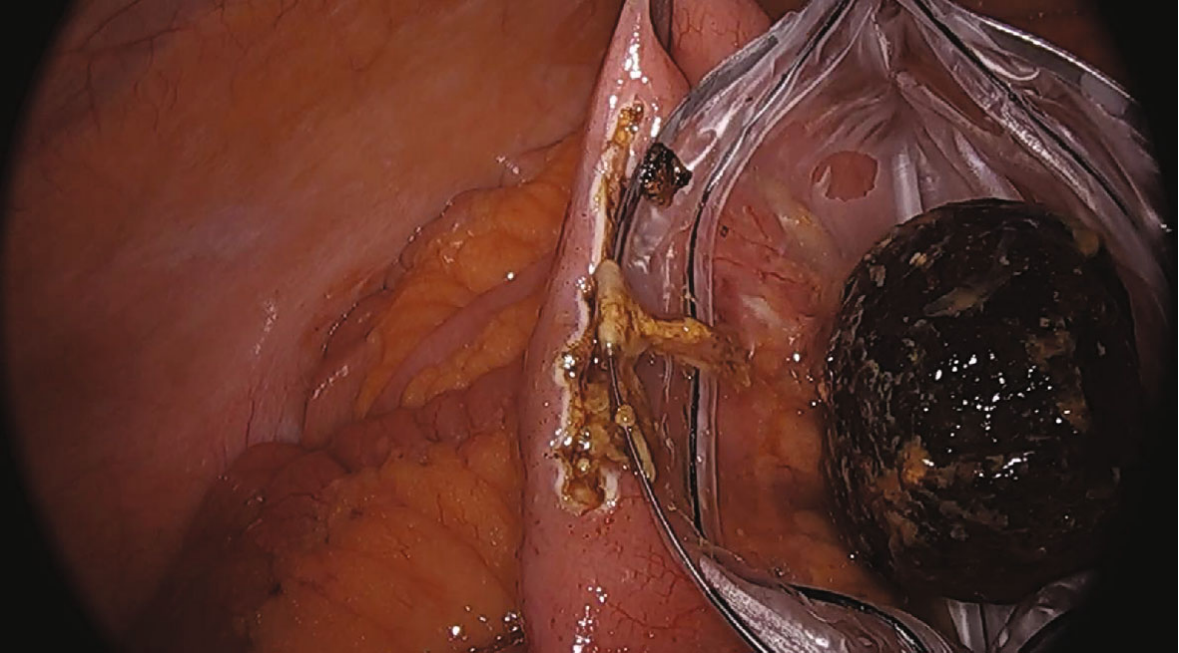
Gallstone placed in endoscopic retrieval bag.

**Figure 4:**
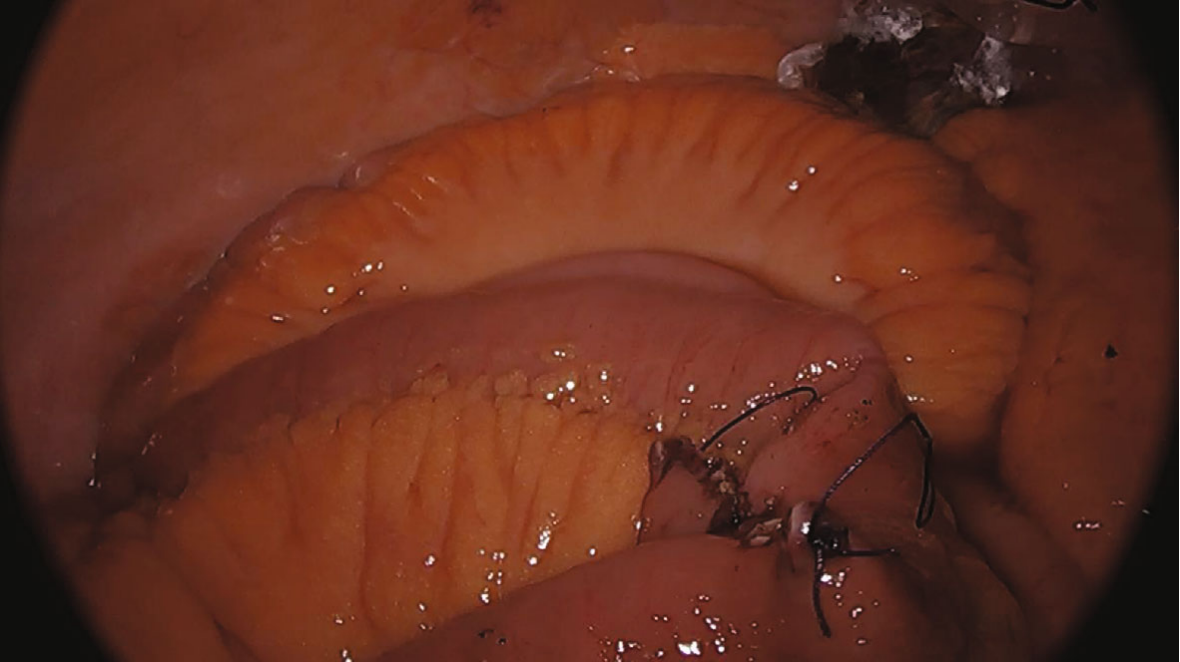
Transverse closure of longitudinal enterotomy.

The small bowel was inspected beginning at the terminal ileum, distal to the obstruction, where it was decompressed, to prevent excessive handling of dilated loops of bowel which could cause iatrogenic enterotomy. The site of obstruction caused by the gallstone was identified at a location several feet proximal to the terminal ileum. Once the obstructing stone was identified, a longitudinal enterotomy was made and the gallstone was extracted, placed in an endoscopic retrieval bag, and removed from the patient. The enterotomy was closed in a transverse fashion using an endoscopic suturing device to avoid narrowing of the small bowel lumen (Figures 2–4), similar to a Heineke-Mikulicz pyloroplasty. A suturing device was used due to its shorter learning curve for residents in comparison to free-hand intracorporeal suturing. Once the stone had been removed and the proximal small bowel was allowed to decompress, the remainder of the small bowel was examined; no further gallstones or obstruction points were identified.

## 3. Results

The patient's postoperative course was complicated by an episode of atrial fibrillation on postoperative day 1, for which cardiology was consulted. She was treated medically with rate control and anticoagulation, which she tolerated well. She was discharged home on postoperative day 2. The patient was seen in clinic for a two-week follow-up visit and was doing well, tolerating a diet, and having regular bowel function. Other than the episode of postoperative atrial fibrillation, there were no other complications.

## 4. Discussion

In the case presented, gallstone ileus was treated laparoscopically, thereby avoiding laparotomy and the prolonged recovery and associated complications. The patient was able to be discharged home on postoperative day 2, which is shorter than the typical postoperative hospitalization period for patients who undergo open management of gallstone ileus.

In the series presented by Halabi et al., the average length of stay for all patients undergoing surgery for gallstone ileus was 12 days; this is longer than the reported laparoscopic and open postoperative stays noted with adhesive bowel obstructions as measured by Lin et al. of 6.4 ± 2.1 and 7.2 ± 2.9, respectively [1, 8]. A review of laparoscopic and open cases for gallstone ileus by Moberg and Montgomery showed a shorter, but not statistically significant, length of stay with laparoscopic procedures, 7 days for laparoscopic versus 10 days for open [8, 9]. When cholecystectomy, fistula closure, and/or bowel resection are undertaken in addition to the enterolithotomy, the operative time and postoperative length of stay for patients were significantly longer [2].

**Figure 5:**
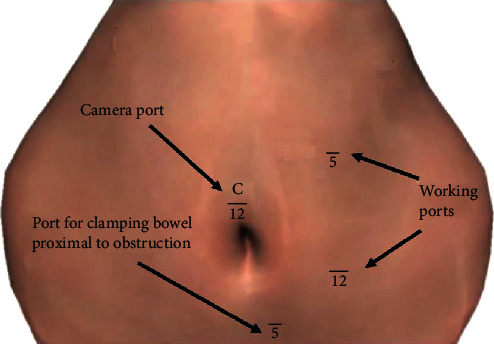
Port placement for gallstone extraction and repair of enterotomy.

A great deal of consideration was taken with regard to the technique for evaluating the small bowel in order to facilitate inspection of its entire length, removal of the gallstone, and subsequent enterotomy closure. Port placement for removing the stone from the distal ileum is depicted in Figure 5. The ports in this position will allow the operating surgeon to examine the distal bowel, remove the gallstone, and close the enterotomy with ease.

**Figure 6:**
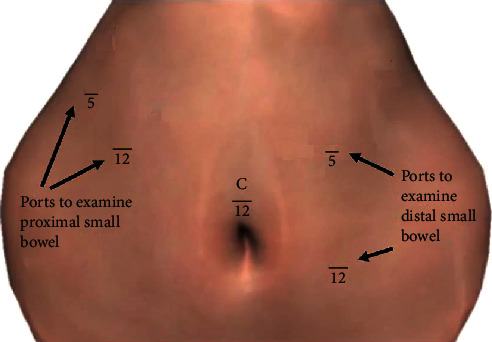
Port placement for laparoscopic examination of small bowel.

In 1997, Drs. Ponsky and Marks published an article describing a technique for laparoscopic examination of the small bowel in an acute setting; their technique uses three ports, one infraumbilical, and one each in bilateral lower quadrants, to examine the small bowel in its entirety [10]. Since that time, there have been few articles published that further address the technical challenge of examining the small bowel in an acute setting. As the use of minimally invasive diagnostic and therapeutic techniques increases within the field of acute care surgery, surgeons must have reproducible minimally invasive techniques that can replicate the open technique of “running the bowel.” Laparoscopic examination of the small bowel was achieved using ports shown in Figure 6. This portconfiguration was created to allow the surgeon to examine the distal half of the small bowel standing on the patient's left side and examine the proximal small bowel standing on the patient's right side.

Furthermore, the use of laparoscopy in adhesive small bowel obstructions has demonstrated a decreased complication risk, including a reduced risk of surgical site infections and death [11]. This is especially important when taking into consideration the increased risk of perioperative complications that patients with obesity have, including ileus and surgical site infection rates [12, 13]. Therefore, the application of minimally invasive techniques on patients with obesity provides improved outcomes, including shorter hospital stay, risk of surgical site infection, and decreased postoperative pain [12].

## 5. Conclusion

A laparoscopic approach for management of gallstone ileus is feasible. The use of laparoscopy for these cases can lead shorter hospitalizations and decreased morbidity when compared to standard laparotomy.

## Supplementary Materials

Supplementary Materials Video depiction of our case study and technique, including port placement and technical approach to running the small bowel, is included.
